# Serum Levels of PCSK9 Are Increased in Patients With Active Ulcerative Colitis Representing a Potential Biomarker of Disease Activity: A Cross-sectional Study

**DOI:** 10.1097/MCG.0000000000001607

**Published:** 2021-09-22

**Authors:** Carla Marinelli, Fabiana Zingone, Maria Giovanna Lupo, Raffaella Marin, Renata D’Incà, Alessandro Gubbiotti, Davide Massimi, Cesare Casadei, Brigida Barberio, Nicola Ferri, Edoardo Savarino

**Affiliations:** * Gastroenterology Section, Departments of Surgery, Oncology, and Gastroenterology; ‡ Medicine, University Hospital of Padua; † Departments of Pharmaceutical and Pharmacological Sciences; § Medicine, University of Padua, Padua, Italy

**Keywords:** cardiovascular risk, ulcerative colitis, PCSK9, inflammation

## Abstract

Ulcerative colitis (UC) is characterized by chronic inflammation and progressive course, with potential extraintestinal complications including cardiovascular mortality. Serum proprotein convertase subtilisin/kexin type 9 (PCSK9) levels have been recently recognized as biomarkers of low-grade inflammation and cardiovascular disease. The aim of our study was to evaluate PCSK9 levels in patients with UC and different degrees of disease activity.

**Methods:**

We prospectively recruited consecutive patients with UC attending our center at the University Hospital of Padua. Demographics, clinical characteristics, and biochemical data, including PCSK9, high sensitivity C-reactive protein, and fecal calprotectin, were recorded. Moreover, endoscopic procedures were performed in all subjects.

**Results:**

We included 112 patients with UC (mean age=52.62±12.84 y; 52.62% males). Patients with UC and abnormal fecal calprotectin (≥250 µg/g) and/or C-reactive protein (≥3 mg/L) had greater levels of PCSK9 compared with UC patients with normal fecal calprotectin and high sensitivity C-reactive protein (*P*=0.03 and 0.005, respectively). Higher endoscopic scores in UC were characterized by greater levels of PCSK9 (*P*=0.03). Furthermore, we found a positive correlation between PCSK9 levels and fecal calprotectin (*r*=0.18, *P*=0.04), endoscopic Mayo Score (*r*=0.25, *P*=0.007), and UC-Riley Index (*r*=0.22, *P*=0.01). We also found a positive correlation between PCSK9 levels and both total and low-density lipoprotein cholesterol values (*P*<0.05).

**Conclusions:**

Serum PCSK9 levels are increased in patients with biochemical and endoscopic evidence of active disease in UC. Further longitudinal studies are necessary to evaluate the role of PCSK9 as a potential biomarker of disease activity and cardiovascular risk in UC.

Ulcerative colitis (UC) is a bowel disease characterized by a chronic inflammation of the gut, localized exclusively to the colon.^1^ The etiology is unknown, with both genetic and environmental factors involved. UC has a progressive course with cumulative intestinal damage and potential development of complications, including extraintestinal manifestations (EIMs).^1^ New evidence suggests that inflammatory bowel diseases (IBDs), particularly UC, are associated with a significant increase of myocardial infarction, stroke, and cardiovascular mortality especially during periods of active disease, although the prevalence of traditional risk factors for cardiovascular disease, such as body mass index (BMI), hypertension, diabetes mellitus, and dyslipidemia is relatively lower in IBD patients than in general population.^2^–^6^

Chronic systemic inflammation plays a crucial role in the progressive course of UC and its complications. Several inflammatory molecules [such as interleukin-1β, interleukin-6, C-reactive protein (CRP)] have been investigated, for their possible pathophysiological role.^7^,^8^ Importantly, the existence of a link between inflammation and hyperlipidemic status has always been recognized, although a common molecular mediator still needs to be identified.^9^ In this context, recent findings have highlighted the association between proprotein convertase subtilisin/kexin type 9 (PCSK9) levels and chronic low-grade inflammation, suggesting their potential role as markers of inflammation and cardiovascular disease. Indeed, PCSK9 is involved in cholesterol homeostasis by posttranscriptional regulating hepatic low-density lipoprotein (LDL) receptor, and for this reason in atherosclerosis. Beyond cholesterol metabolism, PCSK9 has been investigated for its potential pleiotropic effects, regulating several genes involved in apoptosis, proliferation, immune response, and inflammation.^10^ To note, 2 fully human monoclonal antibodies targeting PCSK9 (evolocumab and alirocumab) have been recently approved as PCSK9 inhibitors and released on the market to reduce levels of cholesterol and, therefore, cardiovascular risk.^11^

Given the lack of data on the role of PCSK9 in patients with IBD and the well-known presence of chronic low-grade inflammation in them, the aim of our study was to evaluate PCSK9 serum levels in patients with UC stratified according to disease activity by objective markers of inflammation.

## METHODS

### Study Population

Ethics Committee of Padua approved this prospective cross-sectional study in May 2019 (protocol number 3312/AO/14). All consecutive patients with UC who presented to our endoscopic service for scheduled activity (ie, surveillance, endoscopic assessment of therapeutic response, etc.) were contacted few days before the colonoscopy to explain the study characteristics and to ask to take part in it. In case of acceptance, they were requested to collect stool specimens immediately before the initiation of bowel cleansing preparation. The nature, duration, and purpose of the study were accurately explained. Before performing study-specific procedures, written informed consent was obtained from each patient enrolled. Inclusion criteria included being older than 18 years and a certain diagnosis of UC, according to international criteria, from at least 6 months. Patients were excluded in case of known pregnancy, diagnosis of acute or chronic liver disease, concomitant or past diagnosis of a chronic immune-mediated inflammatory disease other than IBD, history of prior colectomy, evidence of a concurrent diagnosis of another currently active erosive gastrointestinal mucosal disease, ongoing therapy with any cholesterol medication (eg, statins, protein kinase C inhibitors Repatha/evolocumab), or refuse to sign the informed consent form.

### Disease Activity Evaluation

The day of the endoscopic examination, always performed by the same endoscopist (E.S.), all patients who agreed to be enrolled underwent clinical assessment. Demographics and clinical information were drawn from outpatient medical records and/or in collaboration with the patient. The severity of symptoms reported by the patients was recorded according to partial Mayo Score (pMS). Furthermore, Mayo endoscopic subscore was determined during colonoscopy (eMS). Total Mayo Score (MS) was calculated considering the sum between clinical and endoscopic score. The disease was classified as inactive (MS≤2), mild (3≤MS≤5), moderate (6≤MS≤10), or severe (MS>10).^12^ Moreover, all patients provided a stool specimen, collected immediately before the start of bowel preparation, for biochemical activity assessment (ie, fecal calprotectin), according to clinical practice. A value of fecal calprotectin ≥250 μg/g was considered abnormal.^13^

Finally, disease activity was histologically determined on biopsies collected, using UC-Riley Index. This score incorporates 6 histologic features, including: acute and chronic inflammatory cell infiltrate, crypt abscesses and architectural irregularities, mucin depletion, and surface epithelial integrity. A 4-point scale was used to classify each feature as none, mild, moderate, or severe.^14^

### PCSK9, High-sensitivity C-reactive Protein (hsCRP), and Lipidic Profile Determination

Blood samples were collected immediately before colonoscopy for PCSK9, hsCRP, and lipidic profile determination. After centrifuge (15 min at room temperature, 1300*g*), serum was transferred to cryovials and stored at −20°C for further analysis.

Total cholesterol and triglycerides were determined with the enzymatic colorimetric methods using cholesterol oxidase/peroxidase aminophenazone and glycerol phosphate oxidase/peroxidase aminophenazone reagents, respectively (Horiba ABX, cat NN° A11A01634, and A11A01640, respectively), and analyzed with Cobas Mira Plus S (ABX Italy). For high-density lipoprotein cholesterol (HDL-C) determination, serum samples were treated with dextran sulfate 500/magnesium chloride for apolipoprotein B–containing lipoproteins precipitation (very LDL, intermediate-density lipoprotein, and LDL, respectively). Then, HDL-C and total cholesterol were quantified. The Friedewald formula was applied for the determination of low-density lipoprotein cholesterol (LDL-C): LDL-C=total cholesterol−HDL-C−(triglycerides/5).

Serum PCSK9 concentrations were measured using a commercial enzyme-linked immunosorbent assay kit (R&D Systems, MN; cat. N° SPC900) able to recognize free PCSK9. Briefly, serum samples were diluted 1:20, according to manufacturer’s instructions, and incubated into a microplate precoated with a monoclonal antibody specific for human PCSK9. A 4-parameter logistic curve-fit was generated to obtain sample concentrations, using GraphPad Prism 5. The minimum detectable concentration was 0.219 ng/mL. Intra-assay and interassay coefficients of variation were 5.4±1.2% and 4.8±1.9%, respectively.

Serum hsCRP concentrations were measured using a commercial enzyme-linked immunosorbent assay kit (apDia, Belgium; cat. N° 740011) able to recognize circulating CRP. Serum samples were diluted 1:1000, according to manufacturer’s instruction, and incubated into a microplate precoated with a monoclonal antibody specific for human CRP. As suggested, sample concentrations were retrieved by generating a linear curve-fit using GraphPad Prism 5. The minimum detectable concentration was ∼0.02 µg/mL. Intra-assay and interassay coefficients of variation ranged 4.1% to 6.9% and 5.8% to 6.3%, respectively. A value of hsCRP ≥3 mg/L was considered abnormal.^15^

### Statistical Analysis

To the best of our knowledge, no studies evaluated the PCSK9 levels in humans according to the grade of intestinal inflammation or more in general in the IBD population. Thus, to estimate the sample size needed for our study, we empirically aimed to detect an increase of at least 25% of the mean PCSK9 levels found in the general population (65.35±30.15) in subjects with active disease as compared with patients in remission.^16^ Accordingly, we calculated that 47 subjects in remission state and 47 in active state (considering a cutoff of 250 μg/g for calprotectin) were necessary to observe such difference, with a power of 80% and a *P*-value of 0.05.

Continuous variables were indicated as mean with SD or median with 25th to 75th percentiles if normally distributed or not respectively, while categorical variables were indicated as frequency. Possible differences between 2 groups were assessed with the independent-samples *t* test or Mann-Whitney test for parametric and nonparametric variables, respectively. Correlation between PCSK9 and the following continuous variables: eMS, pMS, UC-Riley Index, fecal calprotectin, BMI (underweight BMI<18.5, normal weight BMI=18.5-24.9, overweight BMI 25-29.9, obese BMI≥30), total cholesterol, HDL-C and LDL-C, and triglycerides, were conducted using the Pearson correlation coefficient *r* or Spearman correlation coefficient (rs) for parametric and nonparametric variables, respectively. Linear regression models adjusted for known cardiovascular risk factors (age, sex, BMI, cholesterol, and smoke)^2^ were used to assess the independent correlation between PCSK9 and markers of disease activity. The receiver operating characteristic curve analysis was used to set the most sensitive and specific serum PCKS9 cutoff in detecting disease activity, CRP, fecal calprotectin, Mayo endoscopic scores, measures of histologic inflammation. Using STATA 11 software for data analysis, *P*-value was considered statistically significant when <0.05.

## RESULTS

### Study Population Characteristics

Among 145 eligible patients contacted, 112 consecutive patients with UC agreed to participate and were enrolled in the present study. The demographic and clinical characteristics of our population have been summarized in Table 1. Fifty-nine (52.6%) patients were males, and the mean age was 52.6±12.8 years. Nine (8%) patients were current smokers, whereas 39 (34.8%) were former smokers. Moreover, we found that about half of our patients (58%) had normal weight, whereas 44 (39.3%) were overweight or obese. Finally, as illustrated in Table 1, comorbidities were quite uncommon likely due to the relatively young age of our population.

**TABLE 1:** Main Characteristics of the Study Population

Variables	Study Population (N=112)
Age [mean±SD (range)] (y)	52.6±12.8 (21.1-81.9)
Sex distribution, n (%)	
Male	59 (52.6)
Female	53 (47.4)
Smoking stratus, n (%)	
Never	64 (57.2)
Smoker	9 (8.0)
Ex-smoker	39 (34.8)
BMI, n (%)	
Underweight (BMI<18.5)	3 (2.7)
Normal weight (18.5<BMI<24.9)	65 (58)
Overweight: BMI (25<BMI<29.9)	38 (33.9)
Obese (BMI≥30)	6 (5.4)
Comorbidities, n (%)	
Prior/current cardiovascular disease	22 (19.6)
Diabetes	4 (3.6)
Prior history of peripheral vascular thrombosis	6 (5.4)
Henoch-Schonlein purpura	1 (0.9)
Localization UC, n (%)	
E1	13 (11.6)
E2	36 (32.1)
E3	63 (56.3)
Immunosupressant therapy ongoing, n (%)	15 (13.4)
5-ASA therapy ongoing, n (%)	98 (87.5)
Biological therapy ongoing, n (%)	
Infliximab	15 (14.3)
Humira	6 (5.4)
Vedolizumab	2 (1.8)
Golimumab	5 (4.5)
History of IBD-related surgery, n (%)	3 (2.7)
Calprotectin [median (25th-75th percentiles)]	139 (60.5-418)
Patients with values <250 μg/g	64 (57.4)
Patients with values ≥250 μg/g	48 (42.6)
hsCRP [median (25th-75th percentiles)]	1.2 (0.2-2.5)
Patients with values <3 mg/L	87 (77.7)
Patients with values ≥3 mg/L	25 (22.3)
Partial Mayo Score, n (%)	
Remission	88 (78.6)
Mild disease	17 (15.2)
Moderate-severe disease	7 (6.2)
Endoscopic Mayo Score, n (%)	
Remission	55 (49.1)
Mild disease	30 (26.8)
Moderate-severe disease	27 (24.1)
UC-Riley Index (mean±SD)	4.6±4.4

5-ASA indicates 5-aminosalicylic acid; BMI, body mass index; hsCRP, high-sensitivity C-reactive protein; IBD, inflammatory bowel disease; UC, ulcerative colitis.

Extension of intestinal disease distribution was as follows: 13 (11.62%) patients had proctitis (E1), 36 (32.1%) patients had left-sided disease (E2), and 63 (56.2%) patients had pancolitis (E3). The majority (87.5%) of patients were taking mesalamine therapy, and 28 (26%) a biologic drug. None of the patients was taking tofacitinib. As to disease activity, 88 (78.6%) patients were considered clinically in remission (pMS<2), but only 50% of them had an eMS compatible with a remission status. The median fecal calprotectin value was 139 µg/g, while the median hsCRP was 1.2 mg/L. Therefore, 48 (42.6%) patients showed biochemical active disease (fecal calprotectin ≥250 μg/g),^13^ while 25 (22.3%) patients showed abnormal hsCRP values (≥ 3 mg/L).^15^

As detailed in Table 2, triglycerides determination resulted pathologic (>150 mg/dL, according to clinical practice) in 13 (11.8%) patients, with a mean value of 101.9 mg/dL. Mean total cholesterol was 211 mg/dL (58.5% patients had >200 mg/dL), mean LDL-C was 134.4 mg/dL (76.6% patients had >100 mg/dL) and mean HDL-C was 56.2 mg/dL (58.9% patients had <60 mg/dL). PCSK9 mean value was 165±57.4 ng/mL.

**TABLE 2:** Lipid and PCSK9 Levels at Baseline

Features	Mean±SD (Range)
Triglycerides	101.9±40.9 (39-274)
Patients with values >150 mg/L [n (%)]	13 (11.8)
Total cholesterol	211±42.5 (119-331)
Patients with values >200 mg/L [n (%)]	65 (58.5)
LDL cholesterol	134.4±40.6 (56.4-253.4)
Patients with values >100 mg/L [n (%)]	86 (76.6)
HDL cholesterol	56.2±15.9 (13-110.4)
Patients with values <60 mg/L [n (%)]	66 (58.9)
PCSK9 (ng/mL)	165±57.4 (71.3-377.07)
Median (25th-75th percentiles)	151.01 (126.1-186.1)

HDL indicates high-density lipoprotein; LDL, low-density lipoprotein; PCSK9, proprotein convertase subtilisin/kexin type 9.

### PCSK9 Determination in Study Population

**FIGURE 1:**
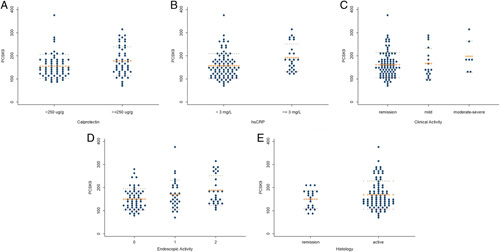
Proprotein convertase subtilisin/kexin type 9 (PCSK9) levels in patients with normal (<250 μg/g) and abnormal (≥250 μg/g) values of fecal calprotectin (A), normal (<3 mg/L), and abnormal (≥3 mg/L) values of high-sensitivity C-reactive protein (hsCRP) (B), in remission, mild, and moderate-to-severe clinical activity according to partial Mayo (C), in remission, mild, and moderate-to-severe endoscopic activity according to Mayo Endoscopy score (D), remission and active histologic inflammation (E).

Table 3 reports PCSK9 levels according to demographics, disease characteristics, treatments, and lipid profiles in our study population. Patients with UC and abnormal fecal calprotectin (≥250 µg/g) and hsCRP (≥3 mg/L) had statistically significant higher levels of PCSK9 compared with UC patients with normal fecal calprotectin and hsCRP (*P*=0.03 and 0.005, respectively). Moreover, higher endoscopic scores in UC were characterized by statistically significant greater serum levels of PCSK9 (*P*=0.03), although PCSK9 values did not differ in our population based on clinical activity (*P*=0.27) (Table 3). Figure 1 shows graphically PCSK9 distribution based on pathologic calprotectin and hsCPR, clinical, endoscopic, and histologic scores.

**TABLE 3:** Determination of PCSK9 Levels According to Demographics, Disease Characteristics, Treatments, and Lipid Profiles in Our Study Population (N=112)

Variables	PCSK9 (Mean±SD)	*P*
Male	159.9±54	0.32
Female	170.7±60.9	
BMI		0.62
Underweight (BMI<18.5)	145.3±64	
Normal weight (18.5<BMI<24.9)	160.9±53.2	
Overweight (25<BMI<29.9)	170.4±55.5	
Obese (BMI≥30)	185.7±106.2	
Calprotectin ≥250 μg/g		0.03*
Yes	177.9±62.6	
No	155.4±51.5	
hsCRP ≥3 mg/L		0.005*
Yes	192.6±60.2	
No	157.1±54.3	
Partial Mayo Score		0.27
Remission	161.9±54.6	
Mild disease	167.6±66.2	
Moderate-severe disease	197.9±67.6	
Severe disease	—	
Endoscopic Mayo Score		0.01*
Remission	150.5±45.9	
Mild disease	170.9±63.2	
Moderate-severe disease	188.1±65.6	
Immunosuppressive therapy		0.66
Yes	159.0±50.9	
No	165.9±58.5	
Biological therapy		0.05
Yes	182.7±67.6	
No	158.9±52.4	
Total cholesterol (>200 mg/dL)		0.35
Yes	170.0±58.6	
No	159.0±56.6	
HDL cholesterol (<60 mg/dL)		0.52
Yes	167.9±55.5	
No	160.9±60.3	
LDL cholesterol (>100 mg/dL)		0.22
Yes	169.1±57	
No	153.5±63.9	
Triglycerides (>150 mg/dL)		0.54
Yes	180.0±59.9	
No	163.5±57.2	

BMI indicates body mass index; HDL, high-density lipoprotein; hsCRP, high-sensitive C-reactive protein; LDL, low-density lipoprotein; PCSK9, proprotein convertase subtilisin/kexin type 9.

*P<0.05.

**FIGURE 2:**
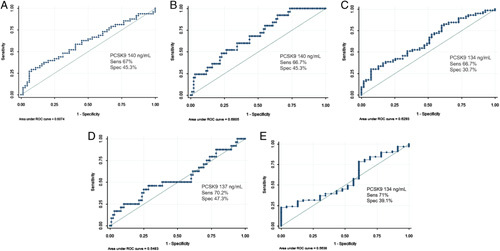
Receiver operating characteristic (ROC) curve analyses for serum proprotein convertase subtilisin/kexin type 9 (PCSK9) in detecting fecal calprotectin (A), high-sensitivity C-reactive protein (B), clinical disease activity (C), Mayo endoscopic scores (D), histologic inflammation (E).

Furthermore, we found a statistically significant positive correlation between PCSK9 levels and fecal calprotectin (*r*=0.18, *P*=0.04), hsCRP (*r*=0.26, *P*=0.006), eMS (*r*=0.25, *P*=0.007), and UC-Riley Index (*r*=0.22, *P*=0.01) in UC patients, as detailed in Table 4. As expected, PCSK9 correlated with total cholesterol and LDL-C levels (*r*=0.28, *P*=0.003 and *r*=0.31, *P*=0.007, respectively). In addition, PCSK9 and HDL-C were negatively correlated (*r*=−0.19, *P*=0.04) (Table 4). Receiver operating characteristic curve analyses for serum PCKS9 in detecting disease activity, hsCRP, fecal calprotectin, eMS were also performed observing limited rates of sensitivity and specificity (Fig. 2).

**TABLE 4:** Pearson and Spearman Correlation Between PCSK9 Levels and Patients’ Characteristics

Features	Spearman or Pearson *r* (vs. PCSK9)	*P*
Endoscopic Mayo	0.25	0.007*
Partial Mayo	0.06	0.49
UC-Riley Index	0.22	0.01*
Calprotectin	0.18	0.04*
hsCRP	0.26	0.006*
BMI	0.10	0.26
Total cholesterol	0.28	0.003*
LDL cholesterol	0.31	0.007*
HDL cholesterol	−0.19	0.04*
Triglycerides	0.20	0.06

BMI indicates body mass index; HDL, high-density lipoprotein; hsCRP, high-sensitivity C-reactive protein; LDL, low-density lipoprotein; PCSK9, proprotein convertase subtilisin/kexin type 9; UC, ulcerative colitis.

*P<0.05.

**FIGURE 3:**
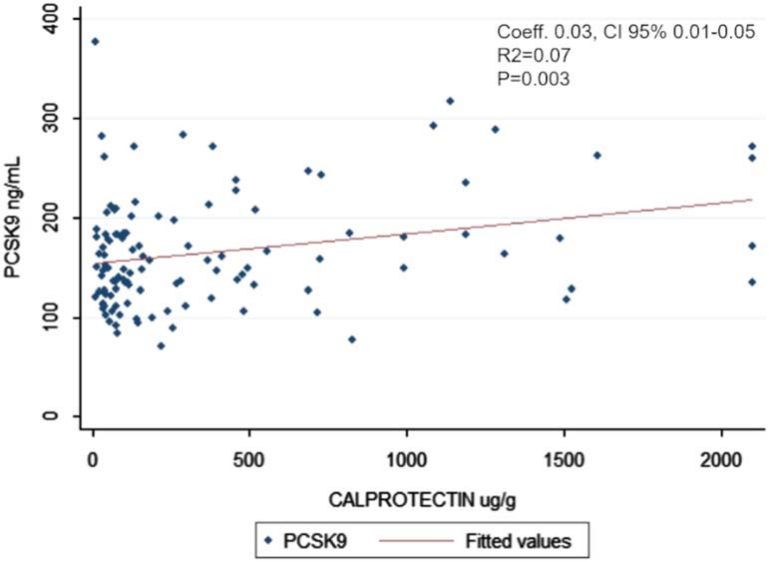
Linear regression model between PCSK9 and calprotectin. CI indicates confidence interval; PCSK9, proprotein convertase subtilisin/kexin type 9.

Linear regression models adjusted for cardiovascular risks factors (age, sex, smoke, BMI, and total cholesterol) confirmed the correlation between PCSK9 and fecal calprotectin (Fig. 3), eMS and UC-Riley Index but not for hsCRP (Supplementary Digital Content 1, http://links.lww.com/JCG/A765).

## DISCUSSION

UC is a chronic condition characterized by periods of recurrence and remission. Indeed, a chronic systemic inflammation despite the lack of symptoms has been demonstrated in previous studies, potentially leading to disease progression and physical as well as psychological disability.^17^–^19^ In addition, patients with UC may experience EIMs that can further modify the natural course of their disease and its morbidity.^20^ PCSK9 has been associated to chronic low-grade inflammation, besides its role in cholesterol metabolism regulation,^10^ and therefore has been recently recognized as a marker of inflammation and cardiovascular risk, with the potential of playing a role of a target for novel therapies.^11^ As well recognized, inflammatory processes are complex and involve several molecules. Patients with UC show increased levels of proinflammatory cytokines, including tumor necrosis factor-α. It has been suggested that tumor necrosis factor-α upregulates PCSK9 mRNA and protein synthesis, determining its circulating levels, as previously demonstrated by Ruscica et al.^21^ Various studies investigated the clinical value of PCSK9 in different conditions, but data in IBD patients are lacking. Thus, we decided to measure the PCSK9 serum levels in patients with UC and to evaluate their correlation with different degrees of disease activity established by clinical, endoscopic, histologic, and biochemical data. We found that serum PCSK9 levels were higher in patients with active UC, and their values were not influenced by confounding factors, including older age, male gender, BMI, and smoking, further supporting the concept that their increasing was related to disease activity.^3^,^22^ Future longitudinal studies are mandatory to confirm the role of PCSK9 in the clinical assessment of UC patients and the value of it as a biomarker of disease activity together with its potential role in the cardiovascular evaluation of these subjects.

Chronic systemic inflammation plays a crucial role in influencing the natural course of inflammatory conditions and the development of EIMs, including the occurrence of cardiovascular events. In order to further explore this relationship, several inflammatory molecules have been investigated in various interventional clinical trials, with however unclear results.^7^ Thus, additional proatherosclerotic pathways have been explored.^23^ In particular, recent findings highlighted the role of PCSK9 levels,^10^ so that novel drugs able to inhibit this protein have been developed and launched on the market after having demonstrated that they had benefit with respect to major adverse cardiovascular events in the trial involving high-risk patients.^24^ Given the increased incidence of cardiovascular events in patients with UC and the similarities with other chronic inflammatory conditions, we investigated the association between PCSK9 and UC. We observed a correlation between serum PCSK9 and fecal calprotectin determination (*r*=0.18, *P*=0.04), a specific marker of intestinal inflammation in IBD patients.^25^ Furthermore, in line with the well-known interaction between inflammation and PCSK9,^26^ we found an important correlation also between PCSK9 and both the eMS and the UC-Riley Histology Index (*r*=0.25, *P*=0.007 and *r*=0.22, *P*=0.01, respectively). To note, our additional analysis (regression analysis) emphasized that PCSK9 values were not influenced by traditional cardiovascular risk factors, and therefore they appeared strictly connected to the inflammatory activity of UC.

We found that PCSK9 levels tended to be higher in IBD female population than in males. Even if this result was not statistically significant, it is in line with previous investigations which observed that hormonal regulation induce PCSK9 overexpression in the female general population.^16^,^27^ Similarly, PCSK9 levels tended to be higher in subjects with higher BMI, as reported in the literature.^28^ We reported PCSK9 values in patients with normal weight (160.9±53.2), in those with clinical and endoscopic remission (161.9±54.6 and 150.5±45.9, respectively) and in those with normal fecal calprotectin and hsCRP (155.4±51.5and 157.1±54.3, respectively) are similar to that recently observed in a normal population with normal weight (156±43 ng/dL).^28^ In contrast, Peng et al^29^ reported a median levels of PCSK9 in 1225 patients with stable cardiovascular disease of 234.52 ng/mL (interquartile levels ranged from 194.79 to 276.13 ng/mL) which is higher compared with those found in our population (median=151.01, interquartile levels ranged from 126.1 to 186.01).

According to medical literature, total cholesterol and LDL-C showed a positive correlation with PCSK9 serum levels, in line with their physiological role.^10^ In addition, increased PCSK9 levels correlated with lower HDL-C levels. According to our results, in a previous meta-analysis encompassing 24 randomized controlled trials including >10,000 patients treated with PCSK9 inhibitors, an HDL-C increase, and an LDL-C reduction compared with the placebo group was demonstrated.^30^

Various studies in medical literature observed that patients with UC present a higher risk of cardiovascular events,^31^–^33^ due to both early atherosclerotic processes and hypercoagulable status likely due to the chronic inflammatory condition.^34^ Indeed, cardiovascular events are more frequently reported in case of disease recurrence or in case of persistent activity.^35^ In a population-based study performed in Olmsted County, Minnesota, from 1980 through 2010, including 736 IBD subjects, Aniwan and colleagues showed, after adjustments for traditional cardiovascular risk factors, that IBD is associated independently with increased risk of acute myocardial infarction [adjusted hazard ratio (aHR), 2.82; 95% confidence interval (CI), 1.98-4.04] and heart failure (aHR, 2.03; 95% CI, 1.36-3.03). Moreover, the relative risk of acute myocardial infarction was significantly increased in patients with Crohn’s disease (CD) (aHR vs. controls, 2.89; 95% CI, 1.65-5.13) or UC (aHR vs. controls, 2.70; 95% CI, 1.69-4.35), whereas the relative risk of heart failure was significantly increased among patients with UC only (aHR, 2.06; 95% CI, 1.18-3.65).^36^ These findings emphasize the need for monitoring cardiovascular risk factors in IBD and their aggressive reduction.^5^ However, to date, no biomarkers of cardiovascular risk have been identified, and future longitudinal studies are mandatory to estimate whether PCSK9 could be adopted for the evaluation of cardiovascular risk also in patients with IBD, as it occurs in patients with cardiovascular diseases.

The strength of this study is represented by its prospective design that permitted us to obtain from each patient clear data regarding clinical and endoscopic disease activity to correlate these features with PCSK9 serum levels. Nonetheless, it is also necessary to highlight an important limitation of the present study design. We carried out a cross-sectional study, so far, follow-up data were not available. A longitudinal study would be more appropriate to evaluate serum PCSK9 fluctuations in parallel with the disease state modifications and the possible cardiovascular complications over time. Of note, we decided to exclude patients with CD because of the marked heterogeneity within this condition, the different molecular characteristics of the 2 IBDs and, the lower risk for cardiovascular events observed in CD patients. However, future studies are needed to evaluate the value of PCSK9 levels measurement also in patients with CD. Third, we have to acknowledge that although we observed a significant correlation between PCSK9 and almost all the variables of disease activity measured, the effect size of the Pearson correlation coefficients were rather small. This could be due to the small sample size or the limited number of patients with a high inflammatory burden. Nevertheless, larger prospective studies are necessary to verify the strength of the association between PCSK9 and disease activity in UC. Finally, although we observed a clear correlation between PCSK9 and fecal calprotectin levels, the spread was wide, thus limiting the value of this observation.

In conclusion, PCSK9 represents an interesting mediator of many cellular processes and inflammatory mechanisms. The present research is the first one in the literature indicating a significant association between elevated PCKS9 serum levels and currently recognized markers of inflammation in UC. Data presented here suggest that PCKS9 serum levels could represent a complementary biomarker of disease activity in UC to be used in parallel with CRP and fecal calprotectin. However, the history of PCSK9 is still evolving, and this study could represent a road map for further investigations in the IBD population aimed to evaluate the role of PCSK9 not only as a biomarker of disease activity but also for cardiovascular risk, together with its therapeutic application for cardiovascular risk reduction.

## Supplementary Material

SUPPLEMENTARY MATERIAL

Supplemental Digital Content is available for this article. Direct URL citations appear in the printed text and are provided in the HTML and PDF versions of this article on the journal's website, www.jcge.com.
