# Supplementary material for: Serum Levels of PCSK9 Are Increased in Patients With Active Ulcerative Colitis Representing a Potential Biomarker of Disease Activity: A Cross-sectional Study
Source: J Clin Gastroenterol. 2021 Sep 22;56(9):787–93. doi: 10.1097/MCG.0000000000001607 (PMC9988229; doi:10.1097/MCG.0000000000001607)
Supplement: SUPPLEMENTARY MATERIAL [file mcg-56-787-s001.doc]

**Supplementary Digital Content Table 1.** Adjusted linear regressions between PCSK9 and markers of disease activity

|  | **Adjusted coeff *** | **r^2^** | *P value* |
| --- | --- | --- | --- |
| Calprotectin | 0.03 (0.01-0.05) | 0.17 | 0.002 |
| hs-CRP | 3.16 (-0.2-6.03) | 0.13 | 0.05 |
| Endoscopic Mayo score | 23.03 (10.4-35.7) | 0.18 | <0.001 |
| UC-Riley Index | 3.8 (1.4-6.2) | 0.16 | 0.002 |

*Adjusted for age, sex, smoke, BMI and total cholesterol
